# Comparison of Child and Adult Mastication of a Sticky Processed Cream Cheese and Simulation with a Masticator

**DOI:** 10.3390/foods13091318

**Published:** 2024-04-25

**Authors:** Coline Caille, Cécile Rannou, Angélique Villière, Clément Catanéo, Araceli Lagadec-Marquez, Julia Bechaux, Carole Prost

**Affiliations:** 1 Oniris VetAgroBio, UMR CNRS 6144 GEPEA, MA(PS)2/USC INRAE 1498 TRANSFORM, 44322 Nantes, France; coline.caille31@gmail.com (C.C.);; 2 Bel Group, Bio-Engineering Team, 41100 Vendôme, France

**Keywords:** processed cream cheese, oral processing, child and adult mastication, masticator, image analysis, texture analysis

## Abstract

An advantage of masticators is the calibration and possible standardization of intra- and inter-individual mastication variability. However, mastication of soft, sticky and melting products, such as processed cream cheeses, is challenging to reproduce with a masticator. The objectives of this work were, for the cheese studied: (1) to compare child and adult mastication and (2) to find in vitro parameters which best reproduce their in vivo chewing. Five parameters influencing mastication (mouth volume, quantity consumed, saliva volume, mastication time and number of tongue–palate compressions) were measured in 30 children (5–12 years old) and 30 adults (18–65 years old) and compared between the two populations. They were then transposed to a masticator (Oniris device patent). The initial cheese, a homogeneous white paste, was surface-colored to investigate its in-mouth destructuring. In vivo boli were collected at three chewing stages (33, 66 and 99% of mastication time) and in vitro boli were obtained by varying the number of tongue–palate compressions and the rotation speed. In vivo and in vitro boli were compared by both image and texture analysis. Child masticatory parameters were proportionally smaller than those of adults. The in vivo child boli were less homogeneous and harder than adult ones. Comparison of in vivo and in vitro bolus color and texture enabled the successful determination of two in vitro settings that closely represented the mastication of the two populations studied.

## 1. Introduction

Mastication is a well-documented oral process during which a lot of complex phenomena occur in a short time: product breakdown via tongue, teeth, palate and jaw actions, saliva impregnation, breathing and swallowing [1,2,3]. Mastication results in a chewed food, named bolus, which is composed of small-size particles moistened by saliva to allow swallowing through the throat without pain and risk of aspiration [4]. This oral process depends on both product and subject; subject could be comprehended through oral status and functions [5]. Indeed, Assad-Bustillos et al. [6] investigated cereal product oral processing of elderly people and highlighted that oral breakdown depended on both the product and the dental status. Devezeaux de Lavergne et al. [7] showed the existence of «short» and «long duration eaters» during sausage consumption, which led to different bolus texture before swallowing, both instrumentally and sensorially. Moreover, mastication, and especially the swallowing point, plays a key role in the release and perception of volatile and taste compounds [1,8]. Investigating and understanding well its mechanisms is therefore of great interest.

During the past decades, a wide range of masticators with different variable parameters were developed to mimic in vivo chewing as faithfully as possible [3,4,9,10,11,12,13]. An advantage of masticators is the standardization of intra- and inter-individual variability. However, it remains challenging to reproduce in a realistic way all the complex and short phenomena which occur during in vivo mastication. In order to best mimic in vivo chewing with the use of a masticator, some in vivo parameters influencing mastication (product quantity, mouth volume, mastication time, saliva rate, etc.) are usually measured and transposed to masticators [14,15,16]. To validate the accuracy of the in vitro reproduction, in vivo and in vitro boli are collected and compared. Bolus collection is usually performed at different chewing stages to be able to follow destructuring kinetics [17,18,19]. Panouillé et al. [20] reviewed the different instrumental methods used to characterize food boli. They include well-known methods such as sieving, rheology, penetrometry, and more recent ones such as tribology and image analysis. If the product studied was initially homogeneous, it could be partly colored before performing image analysis [21,22]. 

Before trying to duplicate in vivo mastication with a masticator, an accurate knowledge of the in vivo process is needed. While in vivo child and adult oral processing have been largely investigated separately [7,14,15,23,24,25,26], they have been little compared and never to our knowledge on processed cream cheese [27,28]. Moreover, there have been studies on in vitro reproduction of breastfeeding infants [29,30], chewing of young and healthy adults [16,31,32] and of elderly people [33,34]. However, to our knowledge, none have focused on the reproduction of a specific population such as children aged between 5 and 12 years old, or even before 20 years old. Therefore, the novelty of this study is that the reproduction of a child panel (5–12 years old), in addition to an adult panel, with a masticator is investigated. Moreover, some in vitro works focused on the reproduction of soft and melting products such as cheeses [32,35,36,37], custard [21], or ice creams [38]. Nevertheless, duplicating the in vivo mastication with a masticator of soft products, such as the processed cream cheese studied in this work, is still challenging since this type of food sticks and melts in the mouth in a short mastication duration. Thus, innovative protocols were set up in this work to be able to deal with a soft, sticky and melting product. 

The aims of the present work are (1) to compare child and adult mastication of the processed cream cheese studied and (2) to find the in vitro parameters which best reproduce in vivo chewing, both for children and adults. The first purpose of this study will offer a deeper understanding of oral processing of children, this in vivo knowledge being necessary to design food which best matches with this specific chewing behavior. Moreover, as a huge inter-individual variability exists within the mastication process [2], the second objective will able to standardize it with a masticator, and thus to improve the repeatability of the in-mouth breakdown. Additionally, a masticator gives access to what happens to food during chewing, i.e., food transformation and bolus formation. Moreover, while in vivo mastication is known to be a complex phenomenon, to investigate it with the aid of a masticator allows the breakdown of the chewing parameters to better understand their impact on bolus characteristics. This deeper mastication understanding can then enable the best adaptation of the product properties to the target population. The parameterized masticator can be used to simulate the release of volatile compounds under consumption conditions.

**Figure 1:**
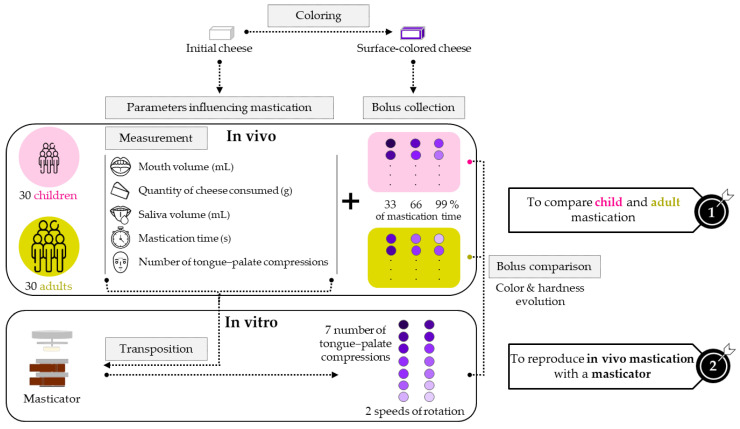
Diagram of the scientific approach.

The scientific approach used in this study is illustrated in Figure 1. The processed cream cheese studied was consumed by children and adults; thus, two panels were recruited to represent both subject categories. Five parameters influencing mastication, usually measured in in vivo and in vitro studies [14,15,16,37], were calculated on these panels and transposed to the masticator (Oniris, 2013, device patent No. 1355509). The five in vitro ratios considered between the cheese sample, the mouth volume and the saliva volume ($\frac{Mouth\;volume}{Quantity\;consumed}$, $\frac{Number\;of\;tongue–palate\;compressions}{Mastication\;time}$, $\frac{Saliva\;volume}{Mastication\;time}$, $\frac{Saliva\;volume}{Mouth\;volume}$, $\frac{Saliva\;volume}{Quantity\;consumed}$) were in accordance with the in vivo ones. We hypothesize that child masticatory parameters are different from those of adults [27]. One of the challenges of this project included working with a soft, sticky and homogeneous cheese paste, which made the usage of well-known characterization methods such as sieving [24,27,37,39] irrelevant. On the contrary, image analysis, which does not require too much handling, seemed to be an efficient method to carry out characterization. The processed cream cheese analyzed, a homogeneous white paste, had to be colored first in order to be able to track its oral destructuring. An innovative way of coloring, only on the surface, was thus performed. With the inner part staying white, uncolored and colored layers were mixed during bolus formation and created a visual indicator of its destructuring degree. Image analysis could then be performed on this heterogeneously colored cheese. Additionally, a more common texture analysis was carried out to characterize hardness evolution through the mastication process. In vivo boli were collected at three different mastication stages (33, 66 and 99% of total mastication time). We hypothesize that the parameters influencing mastication of individuals have an impact on the cheese bolus characteristics [27,40]. In addition, we hypothesize that the mastication of a processed cream cheese by children and adults can be reproduced in vitro using a masticator. In vitro boli were collected at different numbers of tongue–palate compressions and speeds of rotation. The color and hardness of both in vivo and in vitro boli were compared through the different mastication stages to best match in vitro settings with in vivo child and adult chewing.

## 2. Materials and Methods

### 2.1. Cheese Samples and Chemicals

Processed cream cheeses were purchased in a supermarket in Nantes (France) between January 2020 and March 2021, so that they were edible, i.e., before the expiry date at the time of analysis. They were composed of milk (51%), cream (33%), water, milk proteins, lactic ferments, salts and carrageenan. They were stored at 4 °C until analysis. A carrot and hibiscus mixture (Shade Grape Blue-HP-183930) with coloring properties was kindly supplied by GNT-EXBERRY^®^ (Mierlo, The Netherlands). Artificial saliva was weekly prepared according to Van Ruth et al. [41] by dissolving in 1 L of purified water (Milli-Q system, Millipore Corp., Molsheim, France), 0.44 g of CaCl_2_·2H_2_O (Merck, Darmstadt, Germany), 0.48 g of KCl (Merck, Darmstadt, Germany), 0.88 g of NaCl (Fluka, Steinheim, Germany), 1.37 g of K_2_HPO_4_·3H_2_O (Panreac, Barcelona, Spain), 2.16 g of porcin mucin (Sigma, St. Louis, MO, USA), 5.21 g of NaHCO_3_ (Merck, Darmstadt, Germany) and 13.00 g of porcin α-amylase (Sigma, St. Louis, MO, USA).

### 2.2. Cheese Coloring

**Figure 2:**
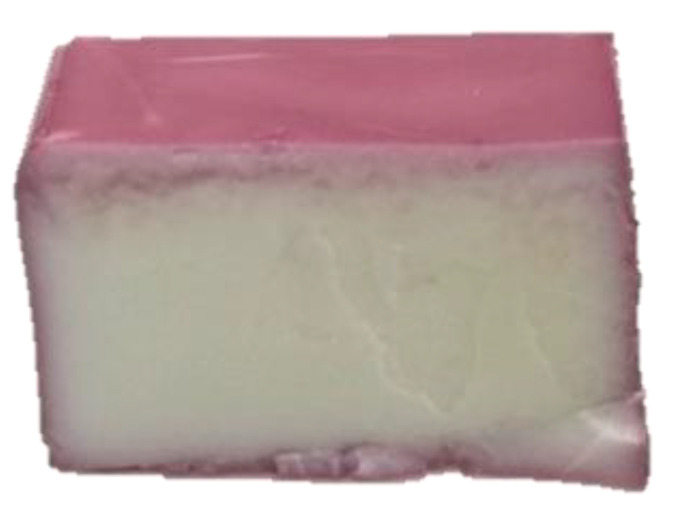
Purple surface-colored cheese.

A purple-colored solution was prepared by dissolving 12.49 g of carrot and hibiscus mixture in 1 L of Evian water, while stirring for 10 min with the aid of a magnetic stirrer. Pieces of cheese (4.5 ± 0.2 g), placed on a fabric fixed in an embroidery frame, were then soaked in the purple solution for 7 min at room temperature. Only the surface of the cheeses was colored; the inner part stayed white (Figure 2). Cheeses were stored at 4 °C in inert transparent glass containers until analysis.

### 2.3. Instrumental Characterizations of the Uncolored and Colored Cheeses

Each instrumental characterization was performed in triplicate. The pH was measured with a pH meter specific to solid product (Mettler Toledo FiveGo, Greifensee, Switzerland). The dry matter was calculated according to the European standard NF EN ISO 55342004-10 [42] for processed cheeses with an oven (FD 240, Binder, Paris, France) at 102 °C for 24 h. A texture analysis, via a penetration test, was adapted from the protocol of Saint-Eve et al. [43]. A traction–compression device (Instron 5544, Instron S.A., Norwood, MA, USA) with a 3 mm diameter cylindrical probe installed on the measurement cell (maximum capacity of 2 kN) was used. A 7 mm penetration was applied to the cheese at a speed of 0.5 mm·s^−1^. Data were recorded using Merlin software (version 5.04., Instron S.A., Norwood, MA, USA). The final charge (N), namely hardness, was extracted from the penetrometry profiles. The color of the cheese surface was measured as explained by Darnay et al. [44] with a chromameter (Konica Minolta CR-400, Tokyo, Japan). The CIE L*a*b* values were recorded (L*, lightness; a*, redness; b*, yellowness). The difference between two colors ΔE was calculated according to the following equation:

$$\Delta E=\sqrt{{\left({\Delta L}^{*}\right)}^{2}+{\left({\Delta a}^{*}\right)}^{2}+{\left({\Delta b}^{*}\right)}^{2}}$$

### 2.4. Sensory and In Vivo Masticatory Analyses

#### 2.4.1. Ethics

The sensory and in vivo masticatory analyses were conducted in accordance with the Declaration of Helsinki and were approved by the ethics evaluation committee of the National Institute of Health and Medical Research in February 2020 (No. 20-657, IRB00003888, IORG0003254, FWA00005831). All applicable institutional and governmental regulations concerning the ethical use of human volunteers were complied with during this research study. Adult panelists and parents of child panelists gave written consent after reading detailed information about the study.

#### 2.4.2. Organization

Panelists were recruited among students and university staff for the adult panel, and by Mérieux Nutrisciences (Saint-Herblain, France) for the child panel. Panelists were instructed not to be pregnant (applicable for women) at the time of analysis, nor smoke, eat, drink coffee or tea, or use any persistent-flavored product in the hour preceding the session. No training was necessary for these sensory tests. The analyses took place in the sensory laboratory of Oniris VetAgroBio (Nantes, France), designed in accordance with international standards (ISO NF EN 85892010-05 [45]). Paper forms were used. Whatever the analysis, panelists rinsed their mouths between each sample with Evian water during a one-minute break. In vivo masticatory analyses with children were performed taking into account the Standard Guide for Sensory Evaluation of Products by Children (ASTM E 2299-11 [46]). Some adaptations were made: ensuring that the analyses were feasible for children aged between 5 and 12, that they were repeatable, and that bolus was expectorated in its entirety, i.e., without too much loss due to uncontrolled swallowing, so that the analysis was representative. 

#### 2.4.3. Triangular Test

This test was carried out to determine if sensory (aroma, taste and texture) differences were perceived in-mouth between the initial uncolored cheese and the colored one. It was performed according to the international standard ISO NF 66582017-11 [47]. Thirty adult subjects participated in a 15 min session, in which they performed two triangular tests (test duplicated). Participants’ eyes were blindfolded so that the subjects could not see the cheese color. Samples (4.5 ± 0.2 g) were served straight from the refrigerator and were given on a spoon. 

#### 2.4.4. Measurements of In Vivo Parameters Influencing Mastication

The processed cream cheese analyzed was consumed by children and adults; thus, two panels were recruited to measure in vivo parameters influencing mastication: panel 1: 30 children, 5–12 years old, 17 girls/13 boys and panel 2: 30 adults, 18–65 years old, 15 women/15 men. The same protocols were used for child and adult measurements. The parameters were measured in triplicate during a 20 min session. At the end of the measurements, age and gender of each subject were asked. The mouth volume and the quantity naturally consumed were measured according to the procedure used by Arvisenet et al. [14]. The mouth volume was measured by asking subjects to take the maximum amount of water into their mouths and spit it out; this amount of water was weighed. The quantity of cheese naturally consumed was measured by asking subjects to bite cheese in a natural way and spit out the bitten piece without chewing it; this bitten cheese piece was weighed. The ratio between the mouth volume and the quantity consumed was then calculated and used to determine the cheese quantity to introduce in the masticator. The mastication time, i.e., the time between the moment subjects put the cheese in their mouths and the moment they swallowed it, and the number of tongue–palate compressions, i.e., the number of vertical masticatory movements, were measured using video recording. The method used was adapted from that of Van Eck et al. [19]. Three stickers were placed on the face of the subjects: one on the forehead to calibrate the video distance and one on the nose and one on the chin to measure the vertical masticatory movements. The subjects had to chew a piece of cheese of 4.5 ± 0.2 g and swallow it in a natural way in front of a camera. The quantity of 4.5 g was chosen as it represents the mean of the quantity naturally consumed by the children, and thus the maximum and same quantity that could be eaten by all the participants (children and adults). Even though multiple swallows could have been considered with this type of food, only the main one was taken into account. The recorded videos were analyzed with Kinovea software (version 0.8.15). Chewing frequency (number of compressions·s^−1^) was then calculated. The saliva content while chewing the piece of cheese was measured according to the procedure used by Repoux et al. [48]. The dry matter (DM) and the water content (WC) of the processed cream cheese and in vivo boli collected just before the swallowing point were measured according to the European standard NF EN ISO 55342004-10 [42] for processed cheeses. The saliva content was calculated for each subject according to the following equation:

$$Saliva\;content\left(\%\right)=\frac{{bolus}_{WC}(\%)}{{bolus}_{DM}(\%)}\times {cheese}_{DM}\left(\%\right)-{cheese}_{WC}(\%)$$

The saliva quantity (=saliva content × 10^−2^ × 4.5, 4.5 g being the weight of the consumed cheese), assimilated to the saliva volume as the saliva is composed of more than 99% water [49], and three ratios were then calculated: $\frac{Saliva\;volume}{Mastication\;time}$ (mL·s^−1^), i.e., the saliva rate; $\frac{Saliva\;volume}{Mouth\;volume}$ (mL·mL^−1^) and $\frac{Saliva\;volume}{Quantity\;consumed}$ (mL·g^−1^).

#### 2.4.5. In Vivo Bolus Collection

The same two panels were used to measure the parameters influencing mastication and to collect the in vivo boli. The subjects were asked to put inside the mouth a colored piece of cheese (4.5 ± 0.2 g), chew it ordinarily, and then spit it out in a petri dish for further characterization. The boli were collected at three different mastication stages as described by Assad-Bustillos et al. [6] or Van Eck et al. [19]: 33% (early mastication), 66% (late mastication) and 99% (moment of swallowing). The times to collect the boli were calculated for each subject according to the individual mastication time previously calculated. As some cheese could be involuntarily swallowed by the panelists while chewing, it was necessary to chew four cheese pieces of 4.5 g by mastication time in order to be able to collect 10 ± 1 g of in vivo boli, which were then transferred to a petri dish. The experiment was realized in duplicate during a 20 min session. 

### 2.5. Masticator and In Vitro Procedure

**Figure 3:**
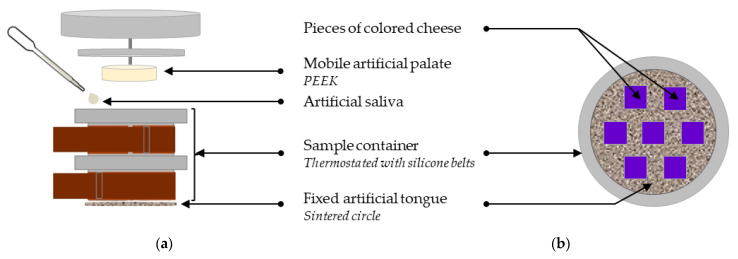
Schematic representation of the masticator. (**a**) Masticator front view. (**b**) The sample container top view with position of the seven pieces of colored cheese.

The masticator used, illustrated in Figure 3a, was composed of a 375 mL container, a sintered circle to reproduce the human tongue, a central plunger with variable speeds of compression (i.e., vertical movement) and rotation (i.e., horizontal movement), and a PolyEtherEtherKetone (PEEK) cylinder (surface of 63.62 cm^2^) fixed on the plunger to reproduce the palate [16]. Indeed, when consuming this type of cheese, it is of great interest to mimic compression movements between tongue and palate. The sample container was maintained at 36 ± 1 °C via hot silicone belts (Vulcanic SAS, Neuilly sur Marne, France) and the temperature was controlled with a thermo tracer (Oceasoft, Montpellier, France) at an acquisition frequency of 1 point·min^−1^. Two hose clamps were used to hermetically close the container.

The bolus destructuring degree being color-controlled, preliminary experiments were carried out to evaluate the role of some in vitro parameters on bolus color evolution. In this way, two variable parameters were selected, namely the number of tongue–palate compressions and the rotation speed. The numbers of tongue–palate compressions were 1, 2, 6, 8, 10, 14 and 18, and the rotation speeds were 4 rpm and 15 rpm. These values were chosen to obtain in vitro mixing states comparable to in vivo ones. The 14 experiments (7 different numbers of tongue–palate compressions × 2 different rotation speeds) were performed in duplicate. A constant cheese quantity of 32 ± 1 g, calculated according to in vivo measurements, was introduced in the masticator in the form of seven cubes (Figure 3b, detail in Section 3.3.1). As the volume of saliva secreted depends on the saliva rate and the mastication progress [50], the different volumes of artificial saliva added in the masticator were calculated according to the in vivo saliva rate and number of tongue–palate compressions (detail in Section 3.3.1). The cheese and the artificial saliva were introduced in the sample container before the mastication process started. After the in vitro mastication, an aliquot of in vitro bolus was collected and 10 ± 1 g of this aliquot was transferred to a petri dish.

### 2.6. Instrumental Characterizations of the In Vivo and In Vitro Boli

#### 2.6.1. Color Evolution Characterization by Image Analysis

As the cheese analyzed was soft, sticky and homogeneous in appearance, the use of well-known characterization methods such as sieving was not relevant. However, image analysis did not require too much handling.

Image acquisition. Images were acquired within 5 min after bolus collection. The imaging system was composed of a camera (Canon PowerShot G10, Cleaver Scientific Ltd., Rugby, UK) equipped with a lens and placed 30 cm above a photographic bench. The lighting system (Oniris VetAgroBio, GEPEA, Nantes, France) was composed of two white-light tubes inside black chamber, thus providing a homogeneous light. As the cheeses were purple surface-colored, images were acquired on green paper (Clairefontaine, 217113E, Top Office, Nantes, France) to emphasize contrast between the product and the background. An image of the top of the petri dish was acquired after texture analysis. Image acquisition parameters were as following: ISO 80, opening: F4.5, shutter speed: 1/50. The pixel images were 2592 × 3456, corresponding to a sample surface of 19.63 cm^2^. This surface was considered to be sufficiently representative of the sample. A total of 360 images were obtained for in vivo cheese breakdown (children and adults) and 28 images for in vitro cheese breakdown with the masticator. 

Image analysis. During the coloring, only the cheese surface was colored; the inner part stayed white. Uncolored and colored layer mixing during mastication created a visual indicator of its destructuring degree. Thus, image analysis was used to quantify this color heterogeneity evolution. The area of interest was extracted from the images to remove the shadows present on the sample. Variance analysis of the red channel was performed on the in vivo and in vitro bolus images. Analysis of variance was carried out as variance is known to be an image marker by measuring pixel intensity variability [51]. The red channel was used instead of the blue or green ones as it maximized the variance of the “child/adult” effect (F = 58.10). The variance of the red channel revealed the bolus homogeneity degree: the higher the red channel variance was, the more the heterogenous the bolus was. The variance of the red channel of child and adult boli was compared, and these results were then compared with those of in vitro boli to find in vitro settings that mimic human chewing in a more realistic way.

#### 2.6.2. Hardness Evolution Characterization by Texture Analysis

Texture analysis was carried out on both in vivo and in vitro boli collected after mastication. A method of back-extrusion type, adapted from that of Aguayo-Mendoza et al. [52], was performed. The destructured product (10 ± 1 g) was placed in a petri dish (55 mm diameter). A cylindrical probe (35 mm diameter) was installed on the measurement cell (maximum capacity of 2 kN). With the use of the petri dish lid (53 mm diameter), a compression was applied at a speed of 0.2 mm·s^−1^ to flatten the bolus until it completely covered the petri dish. Data were recorded using Merlin software (version 5.04., Instron S.A., Norwood, MA, USA). Final charge (N), namely hardness, was extracted from the data. Hardness of child and adult boli was compared, and these results were then compared with those of in vitro boli in order to find the in vitro parameters that best reproduce human mastication.

### 2.7. Statistical Analyses

The statistical analysis for the triangle test was based on the binomial law. A one-way analysis of variance (ANOVA) was performed to compare uncolored and colored cheese instrumental data: Y = “uncolored/colored cheese” (fixed effect). A mixed two-way ANOVA was carried out to compare child and adult masticatory parameters: Y = “subject (child/adult)” (random effect) + “child/adult” (fixed effect). A mixed three-way ANOVA with interaction was applied on the in vivo bolus images and texture measurements to determine if significant mastication differences existed between the children and the adults and over time: Y = “subject (child/adult)” (random effect) + “child/adult” (fixed effect) + “mastication progress” (fixed effect) + “child/adult × mastication progress”. For all the ANOVA analyses, the type III square sum values were taken. For all the tests, a *p*-value of 5% (type I error) was used. ANOVAs were performed using Statgraphics Centurion 19 software (Statpoint Technologies, Warrenton, VA, USA) and R software (version 4.1.1, R Core Team 2021, package: lme4 version 1.1-27). 

## 3. Results and Discussion

### 3.1. The Coloring Did Not Change the Characteristics of the Initial Cheese

#### 3.1.1. Instrumental Level

Food coloring was performed to be able to follow the different destructuring states through the mastication process of the white, homogeneous cheese studied. Nevertheless, it was important to make sure that the coloring did not change the initial properties of the product. Table 1 gives physico-chemical characteristics of the uncolored and colored cheese and the one-way ANOVA results for “uncolored/colored” effect. Food coloring did not change the pH, the dry matter or the hardness of the processed cream cheese studied (*p* > 0.05). This result indicated that the colorant chosen and the way of coloring were suitable for this processed cream cheese. Moreover, the color difference ΔE between the uncolored (reference) and the colored cheese was 42.24. Mokrzycki et al. [53] explained that two different colors can be noticed by observers when ΔE > 5. Thus, the color difference between the initial uncolored cheese and the purple colored one could be detected by human vision.

#### 3.1.2. Sensory Level

To determine if coloring modified the initial sensory characteristics of the cheese, two blind triangular tests were performed (Table 2). No sensory differences were observed between the uncolored and colored cheese, which is in line with the previous instrumental results. Thus, the coloring applied did not seem to change the initial properties of the product, both on an instrumental and a sensory level. Masticatory analyses could therefore be carried out on colored cheese.

### 3.2. Child and Adult Processed Cream Cheese Mastication Presented Some Differences

#### 3.2.1. In Vivo Parameters Influencing Mastication

To investigate child and adult chewing, in vivo parameters influencing mastication were measured. Significant differences were observed for five of the in vivo parameters (Table 3). 

As expected, child mouth volume and quantity consumed were significantly lower than adult ones. The smaller quantity of cheese consumed by children could be linked to their smaller mouth volume. However, there was no significant difference in the ratio $\frac{Mouth\;volume}{Quantity\;consumed}$, which means that the differences observed between the children and the adults regarding the mouth volume and the quantity consumed were proportional, which seems logical. 

Even if no “child/adult” effect was observed on the number of tongue–palate compressions, such an effect was present on the mastication time and the chewing frequency. Children chewed the cheese for less time than adults but with a higher chewing frequency. Child short mastication time could be attributed to the small quantity consumed. Regarding adult mastication, our findings at the swallowing point (mastication time = 8.91 ± 3.31 s; number of compressions = 10.08 ± 3.90; and chewing frequency = 1.16 ± 0.24 compression·s^−1^) are in good agreement with Aguayo-Mendoza et al.’s [52] recent results while working on processed cheeses. Indeed, they observed the following results at swallowing in young healthy adults (average age of 23 years old): mastication time = 10.9 ± 0.4 s, number of chews = 13.3 ± 0.8 and chewing frequency = 1.22 chew·s^−1^. However, another work on model cheeses found higher mastication times (from 12.6 to 28.6 s) and a higher number of chews (from 17.0 to 40.9) at the swallowing point for people with an average age of 41 years old [54]. This could be due to harder products, and, thus, longer mastication times and a higher number of chews. Regarding child mastication, to our knowledge, there were no studies which focused on cheeses. However, while working on raisins and graham crackers, Gisel et al. [26] observed child chewing frequencies (1.19 ± 0.15 chews·s^−1^ and 1.22 ± 0.13 chews·s^−1^, respectively) close to our (1.29 ± 0.23 compressions·s^−1^).

Significant differences between children and adults were observed for the saliva volume but not for the saliva rate. Indeed, the child saliva volume was significantly lower than the adult one but as their mastication time was also lower, their saliva rate was comparable to the adult one. Child lower saliva volume could be associated with child smaller cheese quantity and shorter mastication time. Concerning human saliva rate, Repoux et al. [48] noticed an important variability between subjects (average age of 40 years old) while chewing processed cheeses. Drago et al. [55] measured a stimulated saliva rate on adults (from 29 to 40 years old) on model dairy products of 0.03 ± 0.01 mL·s^−1^. This result, close to ours (0.06 mL·s^−1^), was also found by Roger-Leroi et al. [56] while healthy adults (average age of 24.9 ± 1.1 years old) chewed a Paraffin piece. Concerning child saliva rate, our finding (0.05 ± 0.04 mL·s^−1^) compares well with that of Leonor et al. [57] who found a stimulated saliva rate mean which varied from 0.003 to 0.048 mL·s^−1^ while children (from 7 to 12 years old) were chewing a Paraffin piece (7 ± 0.1 g). Regarding the ratios $\frac{Saliva\;volume}{Mouth\;volume\;}and\;\frac{Saliva\;volume}{Quantity\;consumed}$, no significant differences were observed between the children and the adults. This result indicates that the volume of saliva secreted seems proportional to the mouth volume and the cheese quantity consumed, similar to what was expected.

These in vivo results suggest that children and adults presented some differences in their parameters influencing mastication, which is in line with previous results [27,28]. Indeed, Julien et al. [27] observed that masticatory performances (maximum bite force, surface and contact occlusal areas) of children (6–8 years old) were lower than those of adults (22–35 years old), which could be linked to a body size (height and weight) increase with age.

After studying some parameters influencing mastication in children and adults, their bolus characteristics through the chewing process (33, 66 and 99% of mastication time) were investigated. The results of the in vivo bolus analyses are divided into two parts: firstly, their characterization by image analysis, and, secondly, by texture analysis.

#### 3.2.2. In Vivo Bolus Homogeneity Degree Characterized by Image Analysis

Image analysis, by calculating the variance of the red channel, revealed the bolus homogeneity degree. A three-way ANOVA was performed to study “subject (child/adult)”, “child/adult”, “mastication progress” and their interaction effects on the variance of the red channel. 

**Figure 4:**
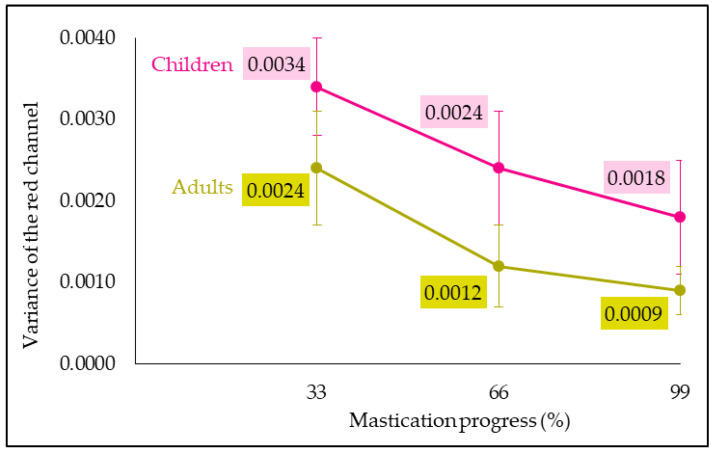
Child and adult red channel variance evolution in function of mastication progress (error bar: Pearson standard deviation).

ANOVA results show that the variance of the red channel significantly decreased over mastication progress (F = 318.88, *p* = 0.0000), revealing an increase in bolus homogeneity degree with chewing time (Figure 4). This result is in good agreement with Prinz et al. [21] and Schimmel et al.’s [58] works in which the mixing degree, also quantified by image analysis, increased with the mastication time and the number of chews, respectively. In addition, Tournier et al. [40], who also used image analysis, observed that the longer the chewing duration, the lower the contrast (image analysis parameter reflecting bread degradation). This tendency could be explained by higher saliva quantity and bolus mixing with mastication time. 

A “child/adult” effect was also observed (F = 58.10, *p* = 0.0000). Bolus red channel variance was higher for children than for adults, revealing a lower homogeneity degree in child boli (Figure 4). The differences observed between children and adults regarding their parameters influencing mastication could explain the differences measured in bolus homogeneity degree. Indeed, the shorter mastication time and lower saliva volume of children could be associated with an in vivo bolus mixing that is less pronounced than that of adults. Such a “child/adult” effect was also found by Julien et al. [27] who observed that girl (6–8 years old) in vivo boli of CutterSil^®^ (i.e., a condensation silicone impression material) had a higher median particle size and distribution broadness than those of women and men (22–35 years old). Within a group of adults, a “subject” effect was noticed by Tournier et al. [40] in a work on bread boli. For a given cycle number, bolus homogeneity varied between the panelists; the higher the mastication efficiency the subject presented, the more the bolus was homogeneous. In our case, the lower mouth volume and the weaker dentition of children could explain the lower bolus homogeneity degree compared to that of adults. 

Figure 4 also reveals that adult red channel variance at 33% was identical to the child one at 66%. The same bolus homogeneity degree was reached at different mastication stages between the two populations studied, at a more advanced stage for children as they destructured the product less than adults.

#### 3.2.3. In Vivo Bolus Hardness Characterized by Texture Analysis

In addition to image analysis, texture analysis was performed on in vivo boli. A three-way ANOVA was performed on texture measurements to study, as for image analysis, both “child/adult” and “mastication progress” effects and their interaction. 

**Figure 5:**
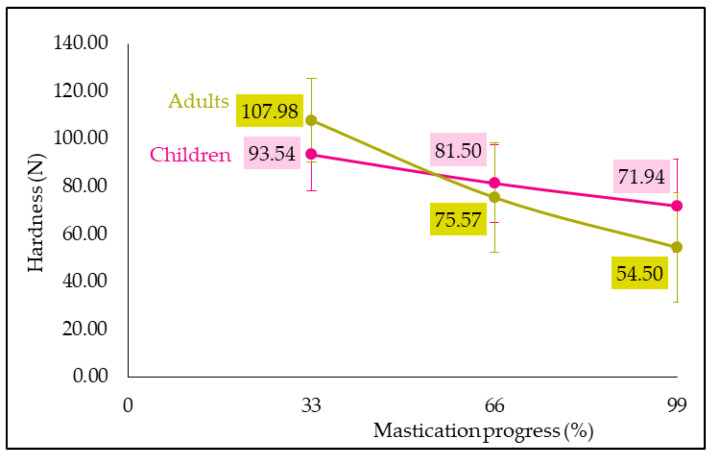
Child and adult hardness evolution in function of mastication progress (error bar: Pearson standard deviation).

As for image analysis, the results demonstrate a “mastication progress” effect (F = 405.36, *p* = 0.0000). The more the mastication progressed, the lower the hardness was, which seems logical (Figure 5). It could be explained by a higher saliva volume and bolus destructuring while chewing progressed, thus leading to a softer texture. This result is in good agreement with Prinz et al. [21] and Raja et al.’s [12] works in which the viscosity and the hardness, respectively, decreased over time as saliva increased.

However, no significant “child/adult” effect was observed on hardness (*p* > 0.05), but the interaction “child/adult × mastication progress” had a significant effect (F = 74.13, *p* = 0.0000). Indeed, as illustrated in Figure 5, the evolution of bolus hardness was different between the two populations. Hardness of child boli decreased less over chewing than the adult ones. An explanation could be that, as adults had lower chewing frequency than children, their boli at 33% of mastication were harder than those of children. In addition, as adults chewed longer than children, they produced more saliva and destructured the product more. Thus, their boli before swallowing were softer than child ones. These texture analysis results suggested that children and adults had different chewing behaviors, which is in good agreement with image analysis results.

#### 3.2.4. In Vivo Measure Discussion

Some of the child masticatory parameters were proportionately lower than those of adults (mouth volume, quantity consumed and saliva volume). For these parameters, children could be seen as small adults, which seems logical since children have not yet completed their growth. However, child boli were more heterogeneous in color and harder in texture than those of adults. This may seem surprising since their parameters influencing mastication were often proportional. There are several possible explanations for the observed differences in cheese boli. One possible explanation is that masticatory parameters other than those presented in the article may be responsible for the observed bolus differences. The number of teeth, for example, could explain the differences. The children in this study had between 19 and 28 teeth, whereas adults in good dental condition, i.e., without dentures and with few artificial crowns and implants, generally have between 28 and 32 teeth, depending on their wisdom teeth. A lower number of teeth in children could be responsible for less-unstructured cheese boli, which were therefore more heterogeneous in color and harder in texture. However, the action of the teeth was less for this type of food, which was soft and melting in the mouth, unless this hypothesis needed to be adjusted. Another, perhaps more plausible, explanation is that masticatory parameters that might be responsible for the observed bolus differences were not measured in this study. Indeed, parameters such as compression force during the bite, muscle work or shear angle could explain the observed bolus differences [25,32,37].

Based on in vivo masticatory analyses, it could be concluded that the mastication of the processed cream cheese studied seemed different between the children and the adults. Young children are usually considered as a specific population regarding oral processing as they have fewer teeth and a smaller oral cavity than adults. In some ways, it is in accordance with a past study in which the in vivo bolus collection method was specifically designed to work with young children [59]. Food feeders, i.e., small mesh bags with a handle, were used. A preliminary test performed on adults showed that one type of feeder gave in vivo boli close to those obtained naturally, without a feeder. This encouraging alternative to collecting in vivo boli may be considered when working with young children. In this work, in vivo boli were collected with the chewing and expectorating method for all the panelists even if there was a wide range of variation between their ages (from 5 to 65 years old). Indeed, the method used turned out to be achievable by both children and adults. 

Moreover, in this study, in vivo boli were collected at 33 and 66% of mastication and just before swallowing (99%), but Jourdren et al. [60] selected the times to collect boli according to Temporal Dominance of Sensations (TDS) curves, 40% being the moment where more differences were perceived between the products. In this study, 33 and 66% were chosen to be able to follow the in vivo destructuring kinetic. Thus, it might not be necessary to study the mastication stages with the higher dominances of sensations. However, selecting chewing stages according to TDS curves could be relevant while studying volatile compound release.

In addition, a single swallow, the main one, was taken into account in this work. However, Aguayo-Mendoza et al. [52] recently observed 2.2 ± 0.2 swallows during the mastication of a processed cheese. Thus, successive swallowing could have been considered as some cheese could remain in the mouth after the first swallow, stuck on the teeth, the palate or the tongue. This in-mouth-remaining food after swallowing, i.e., the mouth-coating, is known to play a role in aroma remanence [48]. Nevertheless, to simulate successive swallowing or mouth-coating with a masticator in a realistic way is not that easy. 

In any event, a clear understanding of the in vivo masticatory process is an important step before trying to duplicate it with a masticator.

### 3.3. Two Ways of In Vitro Mastication Were Therefore Set Up to Better Reproduce the In Vivo Process

#### 3.3.1. Transposition of the In Vivo Parameters Influencing Mastication to the Masticator

As child and adult mastication were different, two ways of in vitro chewing had to be found. To perform such reproduction, in vivo parameters influencing mastication previously measured were transposed to the masticator (Table 4).

Some in vitro parameters were fixed, such as the mouth temperature which was set at 36.00 ± 1.00 °C, in accordance with in vivo measures [61]. The masticator used was designed with a fixed mouth volume of 375.00 mL. As no significant differences were observed regarding the ratio $\frac{Mouth\;volume}{Quantity\;consumed}$ between children and adults, the overall mean (11.47 mL·g^−1^) was considered to determine the cheese quantity to introduce in the sample container, which was thus the same for the two populations (32.69 ± 1.00 g, =375.00/11.47). As for in vivo bolus collection, cheese pieces of 4.5 ± 0.2 g were used; seven pieces of 4.5 g were necessary to obtain a final cheese weight of 32 ± 1 g. As no significant differences were observed on saliva rate between children and adults, a constant saliva rate of 0.05 mL·s^−1^ (overall mean) was used. It was considered from preliminary experiments that the volume of saliva secreted increases linearly with the number of tongue–palate compressions and the pieces of cheese. Thus, the in vitro saliva volume added in the sample container before the mastication process began was as following (in vivo saliva rate × 7 cheese pieces × the number of tongue–palate compressions): 0.35 mL for 1 compression, 0.70 mL for 2 compressions, 2.10 mL for 6 compressions, 2.80 mL for 8 compressions, 3.50 mL for 10 compressions, 4.90 mL for 14 compressions and 6.30 mL for 18 compressions. In this study, the same composition of artificial saliva was used for children and adults (Van Ruth et al. [41]). Human saliva, with a composition close to that of Van Ruth but more complex [49], is known to vary a lot according to the moment of the day and the subjects. Indeed, Ben-Aryeh et al. [62] showed that the composition of child saliva could differ from that of adults. These researchers observed a significant linear ascending correlation with age for concentration of sodium, total protein, immunoglobulin A and amylase activity. To overcome these intra-day and inter-individual variabilities, a standardized artificial saliva was used, which is a common way in in vitro studies. However, it might have been relevant to carry out biochemical analyses (protein concentration, enzyme activity, amount of sodium and potassium, etc.) to characterize the subject saliva to best adapt the formula to the age of the individuals. Some investigations were carried out to compare the impact of human or artificial saliva on the bolus properties. Using a simple water solution to mimic human saliva could be too streamlined. Indeed, Poinot et al. [9] observed differences between in vitro bread boli chewed with water (bolus stickier, with a smoother surface) and with Van Ruth saliva (possible α-amylase action on bread amylose chains). Prinz et al. [21] showed that α-amylase had an important role on the viscosity of a starch-based product by its starch degradation effect. Even working with a non-starch-based product (cream-style dressing), Odake et al. [63] observed that the release of volatile compounds such as butane-2,3-dione decreased with the addition of any ingredient to the water (pH effect). In addition, Roger-Leroi et al. [56] showed that a similar artificial saliva to the one used in this study had a viscosity comparable to that of human saliva. Therefore, the artificial solution employed in this work seemed appropriate to mimic in a realistic way human saliva.

In parallel with these fixed in vitro parameters, the number of tongue–palate compressions and the rotation speed were variable. These two parameters were chosen to vary because, according to exploratory experiments, they were found to be the most impactful on the bolus destructuring state. Their variation ranges were selected to obtain in vitro mixing states that could be compared with the in vivo ones. These two variable parameters are analogous to the ones found to have the more impact on the particle size distribution of in vitro peanut boli [39]. The number of chews was also an efficient in vitro parameter to reduce the particle size of peanut and carrot [15]. Arvisenet et al. [14] noticed the importance of the rotation movements to allow a correct in vitro destructuring state of apples. However, the rotation speeds used in our study (4 and 15 rpm) were slightly lower than the ones used by Arvisenet et al. [14] (10 and 50 rpm), Poinot et al. [9] (17 and 63 rpm) and Guilloux et al. [16] (0, 25, 50 and 75 rpm). The higher rotation speeds applied in these in vitro studies were maybe due to the harder texture of the products analyzed (apple, bread and pizza, respectively), and the reproduction of an adult population with normal healthy dentition and not a child one which can present chewing deficiencies. Additionally, in similar in vitro works, other parameters were studied, still with the objective to best duplicate the in vivo phenomenon: mastication time [21]: 1, 5 and 10 s), chew speed (Guilloux et al. [16]: 18, 45 and 81 cpm), shearing angle value (Mielle et al. [37]: 0, 1/8 h, 1/4 h tooth) or masticatory force (Mielle et al. [37]: 24, 29 and 34 daN; Mishellany-Dutour et al. [31]: 35 to 337 N). Thus, the masticators developed during these last decades present a wide range of variable parameters to best replicate the in vivo complex phenomenon. 

#### 3.3.2. In Vitro Parameters Which Best Reproduce In Vivo Mastication According to Image Analysis

**Figure 6:**
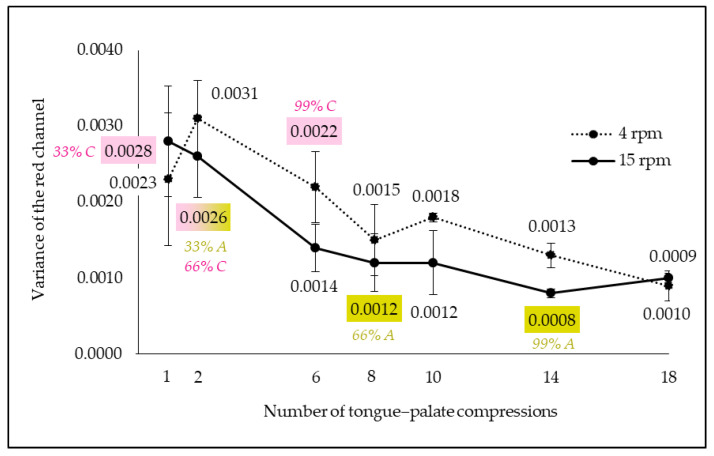
In vitro bolus red channel variance evolution in function of compression number and rotation speed (A/green text and filling: adults; C/pink text and filling: children; pink + green filling: applicable for children and adults; 33, 66 and 99%: mastication progress; error bar: Pearson standard deviation).

The different in vitro boli obtained by varying the number of tongue–palate compressions and the rotation speed were photographed. The variance of the red channel was analyzed on these images (Figure 6). The variance of the red channel decreased with increasing number of compressions, which means that the bolus homogeneity degree increased with mastication progress. It could be explained by higher saliva quantity and bolus destructuring with chewing, thus leading to a higher bolus homogeneity degree. Moreover, the red channel variance of the in vitro boli obtained at 4 rpm was higher than the one obtained at 15 rpm. In other words, the higher the rotation speed, the more homogeneous the bolus. It could be attributed to a more pronounced mixing with higher rotation speed, and thus a higher bolus homogeneity degree. The variance of the red channel of the in vitro boli was compared to that of in vivo boli. This comparison led to the selection of some in vitro parameters to reproduce both child and adult chewing (Table 5). A higher number of tongue–palate compressions was needed to mimic adult mastication than that of children. This result matched with the fact that adult mastication time was higher than that of children.

#### 3.3.3. In Vitro Parameters Which Best Reproduce In Vivo Mastication According to Texture Analysis

**Figure 7:**
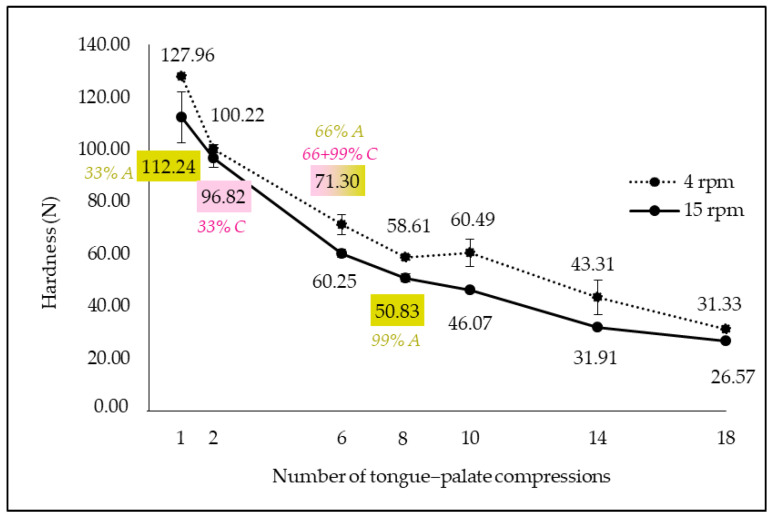
In vitro bolus hardness evolution in function of compression number and rotation speed (A/green text and filling: adults; C/pink text and filling: children; pink + green filling: applicable for children and adults; 33, 66 and 99%: mastication progress; error bar: Pearson standard deviation).

In parallel with image analysis, texture measurements were performed on in vitro boli. The impact of number of tongue–palate compressions and rotation speed on in vitro bolus hardness was studied (Figure 7). Bolus hardness decreased with an increasing number of compressions. This could be explained by higher saliva quantity and bolus destructuring with chewing, thus leading to softer boli. This result is comparable to that of Raja et al. [12], who observed that the hardness of in vitro boli decreased with mastication time. Moreover, hardness of the in vitro boli obtained at 4 rpm was higher than that obtained at 15 rpm. The higher the rotation speed, the softer the bolus was. This result could be attributed to a higher product destructuring with higher rotation speed, and thus softer boli. Hardness of the in vitro boli was compared with that of in vivo boli and some in vitro settings were selected to reproduce both child and adult mastication (Table 6). A higher number of tongue–palate compressions was needed to imitate adult mastication than child chewing, which is in line with image analysis results.

#### 3.3.4. Final In Vitro Parameters Selected

Overall, image and texture analyses showed similar results and trends: higher number of tongue–palate compressions led to lower red channel variance and hardness; lower rotation speeds led to higher red channel variance and hardness; higher compression numbers needed to be applied to best imitate adult mastication in comparison with child mastication. Six tongue–palate compressions were enough to reproduce child chewing until swallowing (99%), according to both image and texture analysis. However, to reproduce adult mastication at swallowing, results differed between image analysis (14 compressions) and texture analysis (8 compressions). These different findings suggested that the cheese hardness might have decreased too quickly during the in vitro mastication compared with what was actually observed in vivo. An explanation could be an excessive setting temperature in the sample container of the masticator (36 ± 1 °C), which did not take into account the lower temperature of the added cheese (4 °C). Indeed, the introduction of a cold product in the oral cavity must have decreased the initial in-mouth temperature. A slightly lower in vitro temperature could have been considered, and, thus, a slower hardness decrease with chewing could have been observed, especially for longer mastication times such as adult ones. This test could be retained in future work. Moreover, the standard deviations of in vitro measurements were quite low, particularly for hardness results and variance of the red channel results for high tongue–palate compression numbers. This result meant that in vitro mastication was repeatable, which was positive. 

In vitro parameters resulting from image analysis were finally chosen to reproduce child and adult in vivo mastication. Image analysis results were chosen rather than texture ones because the bolus mixing state seemed more important than hardness to best mimic in vivo chewing [11,21,40,58]. Table 7 summarizes the in vitro settings that best reproduce both child and adult mastication at the three chewing stages (33, 66 and 99% of mastication). The in vitro settings which best reproduce the in vivo swallowing point (99%), key chewing stage in flavor release and perception [1], were “6 compressions—4 rpm” for children, and “14 compressions—15 rpm” for adults. Arvisenet et al. [14] found comparable rotation speed (10 rpm) to mimic apple adult chewing. Thus, masticators provide a wide range of parameter variation in order to best adapt the chewing duplication to the product or to the population studied. If the masticator used in this work presents numerous functionalities, it can be noted that its tongue movement is limited. Developing tongue rotation, for example, could be a room for improvement as the tongue plays an important role in the chewing process, especially with soft and sticky product like the processed cream cheese studied.

## 4. Conclusions

Building upon previous works on adult mastication, this project further explores and characterizes the mastication of young children, a specific population less investigated. Moreover, this study enables a deeper understanding of the in vivo mastication of a soft product represented by a processed cream cheese, for both children and adults, with the end goal to be able to design food that best matches with specific segments of the population.

The measurement of parameters influencing mastication in 30 children (5–12 years old) and 30 adults (18–65 years old) shows that, overall, child masticatory parameters are significantly smaller than those of adults, but no significant differences are observed regarding the ratios. The color of in vivo child boli is less homogeneous and their texture is harder than adult ones. Thus, the results of in vivo masticatory analyses suggest that the two populations studied have different masticatory behaviors. However, at the end of this study, it remains difficult to explain the bolus differences observed between children and adults by the parameters influencing mastication measured. In future, other parameters will need to be measured to better understand the bolus differences. Appropriate instrumental methods to work with homogeneous, soft and sticky cheese boli have been carried out, particularly cheese coloring and image analysis. Additionally, in vivo boli have been successfully collected at three mastication stages (33, 66 and 99% of chewing) with the chewing and expectorating method, for either children or adults. This work is the first investigation to compare child and adult mastication of a processed cream cheese. The in vivo parameters influencing mastication calculated in this project could be used in future in vivo and in vitro studies with the objective to best adapt the in vitro mastication to the product or the population studied.

The masticator parameters that best reproduce in vivo mastication, for both children and adults, have been successfully set up at the three mastication stages (33, 66 and 99% of chewing). In this way, this study provides the first work to duplicate child mastication with the use of a masticator. The masticator used in this work is therefore a powerful tool to mimic the in vivo mastication of a soft, sticky and melting product of a specific population in a realistic way. However, it remains difficult to faithfully reproduce in vivo tongue movements using in vitro tools. In future, it may be worthwhile to think about a new geometry or new movements for the in vitro tongue. At present, the in vitro parameters are set up to reproduce both child and adult mastication of the processed cream cheese studied. A future work could be to investigate the impact of cheese properties, such as the texture, on the in vitro bolus destructuring state and, thus, on the volatile and taste compound release.

## Figures and Tables

**Table 1:** Mean ± Pearson standard deviation of the instrumental characteristics of the uncolored and colored cheese (triplicates) and ANOVA results (α = 5%; *** *p* < 0.001).

Variables	Uncolored Cheese	Colored Cheese	Fisher	*p*-Values
pH	5.46 ± 0.33	5.42 ± 0.01	2.57	0.1841
Dry matter (%)	42.97 ± 0.24	43.15 ± 0.24	0.60	0.4805
Hardness (N)	0.66 ± 0.02	0.70 ± 0.01	6.26	0.0666
Chromametric values	L* = 90.81 ± 0.15	L* = 57.01 ± 1.87	391.06	0.0003 ***
	a* = −1.67 ± 0.02	a* = 19.82 ± 0.77	926.42	0.0001 ***
	b* = 11.38 ± 0.23	b* = −2.04 ± 0.25	2244.89	0.0000 ***
	ΔE_colored-uncolored_ = 42.24			

**Table 2:** Results of the two blind triangle tests performed on the uncolored and colored cheeses (α = 5%).

Tests	Number of Panelists *	Number of Correct Answers of the Test	Number of Correct Answers Necessary to Have Significant Difference	Significant Difference between the Samples
Triangular test No. 1	30	5	16	No
Triangular test No. 2	30	9	16	No

* The same panelists performed the two triangular tests.

**Table 3:** Mean ± Pearson standard deviation of the in vivo parameters influencing mastication (triplicates) and ANOVA results for “child/adult” effect (α = 5%; * *p* < 0.05; ** *p* < 0.01; *** *p* < 0.001).

Variables	Children (×30)	Adults (×30)	Fisher	*p*-Values
Mouth volume (mL)	38.24 ± 14.19	84.75 ± 17.71	129.29	0.0000 ***
Quantity consumed (g)	4.52 ± 2.11	7.39 ± 2.44	25.75	0.0000 ***
$\frac{Mouth\;volume}{Quantity\;consumed}$ (mL·g^−1^)	10.27 ± 6.46	12.68 ± 4.78	3.14	0.0816
Mastication time (s)	6.92 ± 3.25	8.91 ± 3.31	6.19	0.0158 *
Number of tongue–palate compressions	8.62 ± 3.78	10.08 ± 3.90	2.40	0.1267
Chewing frequency (compressions·s^−1^)	1.29 ± 0.23	1.16 ± 0.24	5.41	0.0235 *
Saliva volume (mL)	0.28 ± 0.23	0.52 ± 0.41	9.57	0.0030 **
$\frac{Saliva\;volume}{Mastication\;time}=$ saliva rate (mL·s^−1^)	0.05 ± 0.04	0.06 ± 0.04	3.19	0.0793
$\frac{Saliva\;volume}{Mouth\;volume}$ (mL·mL^−1^)	0.01 ± 0.01	0.01 ± 0.01	2.05	0.1580
$\frac{Saliva\;volume}{Quantity\;consumed}$ (mL·g^−1^)	0.08 ± 0.10	0.08 ± 0.07	0.01	0.9366

**Table 4:** In vivo parameter transposition to the masticator (ANOVA results for “child/adult” effect on in vivo parameters influencing mastication; α = 5%; *** *p* < 0.001; NA: Non-Applicable).

Parameters Influencing Mastication		Children	Adults	*p*-Values	Masticator
Fixed	Mouth volume (mL)	38.24 ± 14.19	84.75 ± 17.71	0.0000 ***	375.00
	Quantity consumed (g)	4.52 ± 2.11	7.39 ± 2.44	0.0000 ***	32.69 ± 1.00 (=375.00/11.47)
	$\frac{Mouth\;volume}{Quantity\;consumed}$ (mL·g^−1^)	10.27 ± 6.46	12.68 ± 4.78	0.0816	11.47 (overall mean)
	Saliva rate (mL·s^−1^)	0.05 ± 0.04	0.06 ± 0.04	0.0793	0.05 (overall mean)
Variable	Number of compressions	8.62 ± 3.78	10.08 ± 3.90	0.1269	1–2–6–8–10–14–18
	Rotation speed (rpm)	NA	NA	NA	4–15

**Table 5:** In vitro parameters selected according to image analysis.

	In Vivo Red Channel Variance	Mastication Progress (%)	In Vitro Red Channel Variance	Corresponding Parameters			
Children	0.0034	33	0.0028	1	compression	–	15 rpm
	0.0024	66	0.0026	2	compressions	–	15 rpm
	0.0018	99	0.0022	6	compressions	–	4 rpm
Adults	0.0024	33	0.0026	2	compressions	–	15 rpm
	0.0012	66	0.0012	8	compressions	–	15 rpm
	0.0009	99	0.0008	14	compressions	–	15 rpm

**Table 6:** In vitro parameters selected according to texture analysis.

	In Vivo Hardness (N)	Mastication Progress (%)	In Vitro Hardness (N)	Corresponding Parameters			
Children	93.54	33	96.82	2	compressions	–	15 rpm
	81.50	66	71.30	6	compressions	–	4 rpm
	71.94	99	71.30	6	compressions	–	4 rpm
Adults	107.98	33	112.24	1	compression	–	15 rpm
	75.57	66	71.30	6	compressions	–	4 rpm
	54.50	99	50.83	8	compressions	–	15 rpm

**Table 7:** Examples of child and adult bolus images at the three mastication stages (33, 66 and 99%) and the corresponding in vitro bolus images with the selected in vitro parameters.

Children	In vivo	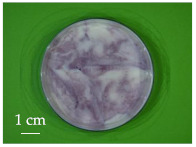	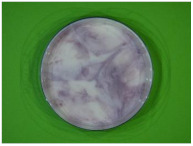	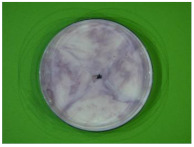
	Mastication progress (%)	33	66	99
	In vitro	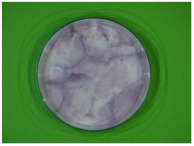	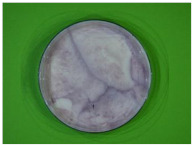	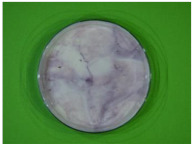
	Settings	1 compression—15 rpm	2 compressions—15 rpm	6 compressions—4 rpm
Adults	In vivo	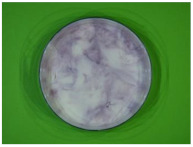	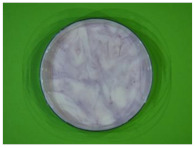	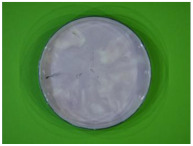
	Mastication progress (%)	33	66	99
	In vitro	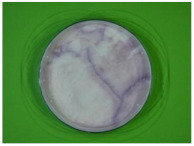	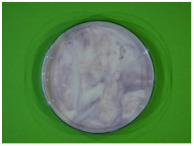	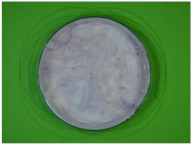
	Settings	2 compressions—15 rpm	8 compressions—15 rpm	14 compressions—15 rpm

## Data Availability

The data presented in this study are available on request from the corresponding author due to data are not public as they were acquired for a private company.

## References

[1] Salles C., Chagnon M.-C., Feron G., Guichard E., Laboure H., Morzel M., Semon E., Tarrega A., Yven C. (2010). In-Mouth Mechanisms Leading to Flavor Release and Perception. Crit. Rev. Food Sci. Nutr..

[2] Feron G., Salles C. (2018). Food Oral Processing in Humans: Links Between Physiological Parameters, Release of Flavour Stimuli and Flavour Perception of Food. Int. J. Food Stud..

[3] Panda S., Chen J., Benjamin O. (2020). Development of model mouth for food oral processing studies: Present challenges and scopes. Innov. Food Sci. Emerg. Technol..

[4] Peyron M.-A., Woda A. (2016). An update about artificial mastication. Curr. Opin. Food Sci..

[5] Ketel E.C., Aguayo-Mendoza M.G., de Wijk R.A., de Graaf C., Piqueras-Fiszman B., Stieger M. (2019). Age, gender, ethnicity and eating capability influence oral processing behaviour of liquid, semi-solid and solid foods differently. Food Res. Int..

[6] Assad-Bustillos M., Tournier C., Feron G., Guessasma S., Reguerre A.L., Della Valle G. (2019). Fragmentation of two soft cereal products during oral processing in the elderly: Impact of product properties and oral health status. Food Hydrocoll..

[7] Devezeaux de Lavergne M., Derks J.A.M., Ketel E.C., de Wijk R.A., Stieger M. (2015). Eating behaviour explains differences between individuals in dynamic texture perception of sausages. Food Qual. Pref..

[8] Wilson C.E., Brown W.E. (1997). Influence of food matrix structure and oral breakdown during mastication on temporal perception of flavor. J. Sens. Stud..

[9] Poinot P., Arvisenet G., Grua-Priol J., Fillonneau C., Prost C. (2009). Use of an artificial mouth to study bread aroma. Food Res. Int..

[10] Morell P., Hernando I., Fiszman S.M. (2014). Understanding the relevance of in- mouth food processing. A review of in vitro techniques. Trends Food Sci. Technol..

[11] Sonmezdag A.S., Cataneo C., Rannou C., Selli S., Prost C. (2022). Elucidation of retro- and orthonasal aroma differences in biscuits (panis biscoctus) using artificial masticator. J. Food Process. Preserv..

[12] Raja V., Priyadarshini S.R., Moses J.A., Anandharamakrishnan C. (2022). A dynamic *in vitro* oral mastication system to study the oral processing behavior of soft foods. Food Funct..

[13] Zhang X., Chen J., Panda S., Benjamin O. (2023). Feasibility analysis of a newly developed multifunctional mastication simulator. Innov. Food Sci. Emerg. Technol..

[14] Arvisenet G., Billy L., Poinot P., Vigneau E., Bertrand D., Prost C. (2008). Effect of Apple Particle State on the Release of Volatile Compounds in a New Artificial Mouth Device. J. Agric. Food Chem..

[15] Woda A., Mishellany-Dutour A., Batier L., François O., Meunier J.-P., Reynaud B., Alric M., Peyron M.-A. (2010). Development and validation of a mastication simulator. J. Biomech..

[16] Guilloux M., Tarancon P., Catanéo C., Vigneau E., Le Bail A., Lethuaut L., Prost C. Efficiency of a new artificial mouth prototype to mimic salt release during food oral processing in order to explain dynamic saltiness perception of pizza varying in salt content. Proceedings of the 3rd International Conference Food Oral Processing.

[17] Peyron M.-A., Gierczynski I., Hartmann C., Loret C., Dardevet D., Martin N., Woda A. (2011). Role of Physical Bolus Properties as Sensory Inputs in the Trigger of Swallowing. PLoS ONE.

[18] Devezeaux de Lavergne M., Tournier C., Bertrand D., Salles C., van de Velde F., Stieger M. (2016). Dynamic texture perception, oral processing behaviour and bolus properties of emulsion-filled gels with and without contrasting mechanical properties. Food Hydrocoll..

[19] Van Eck A., Hardeman N., Karatza N., Fogliano V., Scholten E., Stieger M. (2019). Oral processing behavior and dynamic sensory perception of composite foods: Toppings assist saliva in bolus formation. Food Qual. Pref..

[20] Panouillé M., Saint-Eve A., Souchon I. (2016). Instrumental methods for bolus characterization during oral processing to understand food perceptions. Curr. Opin. Food Sci..

[21] Prinz J.F., Janssen A.M., de Wijk R.A. (2007). In vitro simulation of the oral processing of semi-solid foods. Food Hydrocoll..

[22] Tournier C., Devezeaux de Lavergne M., Van de Velde F., Stieger M., Salles C., Bertrand D. (2017). Investigation of oral gels breakdown using image analysis. Food Hydrocoll..

[23] Castelo P.M., Pereira L.J., Bonjardim L.R., Gavião M.B.D. (2010). Changes in bite force, masticatory muscle thickness, and facial morphology between primary and mixed dentition in preschool children with normal occlusion. Ann. Anat.-Anatomischer Anzeiger..

[24] Marquezin M.C.S., Kobayashi F.Y., Montes A.B.M. (2013). Assessment of masticatory performance, bite force, orthodontic treatment need and orofacial dysfunction in children and adolescents. Arch. Oral Biol..

[25] Scudine K.G.O., de Moraes K.N., Miyagui S.A., Lamy E., Lopes M.F., Mamani M.H., Castelo P.M. (2023). Understanding the relationship between orofacial structures and feeding habits of preschoolers: A multivariate analysis. J. Texture Stud..

[26] Gisel E.G. (1988). Chewing cycles in 2- to 8-year-old normal children: A developmental profile. Am. J. Occup. Ther..

[27] Julien K.C., Buschang P.H., Throckmorton G.S., Dechow P.C. (1996). Normal masticatory performance in young adults and children. Arch. Oral Biol..

[28] Hama Y., Hosoda A., Kubota C., Guo R., Soeda H., Yamaguchi K., Okada M., Minakuchi S. (2022). Factors related to masticatory performance in junior and senior high school students and young adults: A cross-sectional study. J. Prosthodont. Res..

[29] Gerrard S.E., Orlu-Gul M., Tuleu C., Slater N.K.H. (2013). Modeling the Physiological Factors That Affect Drug Delivery from a Nipple Shield Delivery System to Breastfeeding Infants. J. Pharm. Sci..

[30] Scheuerle R.L., Kendall R.A., Tuleu C., Slater N.K.H., Gerrard S.E. (2017). Mimicking the Impact of Infant Tongue Peristalsis on Behavior of Solid Oral Dosage Forms Administered During Breastfeeding. J. Pharm. Sci..

[31] Mishellany-Dutour A., Peyron M.-A., Croze J., François O., Hartmann C., Alric M., Woda A. (2011). Comparison of food boluses prepared in vivo and by the AM2 mastication simulator. Food Qual. Pref..

[32] Tarrega A., Yven C., Semon E., Mielle P., Salles C. (2019). Effect of Oral Physiology Parameters on In-Mouth Aroma Compound Release Using Lipoprotein Matrices: An In Vitro Approach. Foods.

[33] Peyron M.-A., Santé-Lhoutellier V., François O., Hennequin M. (2018). Oral declines and mastication deficiencies cause alteration of food bolus properties. Food Funct..

[34] Hayashi K., Nakada Y., Sémon E., Salles C. (2022). Retronasal Aroma of Beef Pate Analyzed by a Chewing Simulator. Molecules.

[35] Lvova L., Denis S., Barra A., Mielle P., Salles C., Vergoignan C., Di Natale C., Paolesse R., Temple-Boyer P., Feron G. (2012). Salt release monitoring with specific sensors in “in vitro” oral an digestive environments from soft cheeses. Talanta.

[36] Meullenet J.-F., Gandhapuneni R.K. (2006). Development of the BITE Master II and its application to the study of cheese hardness. Physiol. Behav..

[37] Mielle P., Tarrega A., Sémon E., Maratray J., Gorria P., Liodenot J.J., Liaboeuf J., Andrejewski J.-L., Salles C. (2010). From human to artificial mouth, from basics to results. Sens. Actuators B..

[38] Ayed C., Martins S.I.F.S., Williamson A.-M., Guichard E. (2018). Understanding fat, proteins and saliva impact on aroma release from flavoured ice creams. Food Chem..

[39] Salles C., Tarrega A., Mielle P., Maratray J., Gorria P., Liaboeuf J., Liodenot J.-J. (2007). Development of a chewing simulator for food breakdown and the analysis of in vitro flavor compound release in a mouth environment. J. Food Eng..

[40] Tournier C., Grass M., Zope D., Salles C., Bertrand D. (2012). Characterization of bread breakdown during mastication by image texture analysis. J. Food Eng..

[41] Van Ruth S.M., Roozen J.P., Cozijnsen J.L., Posthumus M.A. (1995). Volatile compounds of rehydrated French beans, bell peppers and leeks. Part II. Gas chromatography/sniffing port analysis and sensory evaluation. Food Chem..

[42] (2004). Cheese and Processed Cheese-Determination of the Total Solid Content.

[43] Saint-Eve A., Panouillé M., Capitaine C., Déléris I., Souchon I. (2015). Dynamic aspects of texture perception during cheese consumption and relationship with bolus properties. Food Hydrocoll..

[44] Darnay L., Vitális F., Szepessy A., Bencze D., Csurka T., Surányi J., Laczay P., Firtha F. (2022). Comparison of different visual methods to follow the effect of milk heat treatment and MTGase on appearance of semi-hard buffalo cheese. Food Control.

[45] (2010). Sensory Analysis-General Guidance for the Design of Test Rooms.

[46] (2011). Standard Guide for Sensory Evaluation of Products by Children.

[47] (2017). Sensory Analysis-Methodology-General Guidance-Part 5.2.3: Triangle Test.

[48] Repoux M., Labouré H., Courcoux P., Andriot I., Sémon É., Yven C., Feron G., Guichard E. (2012). Combined effect of cheese characteristics and food oral processing on in vivo aroma release: In Vivo aroma release during cheeses oral processing. Flavour Fragr. J..

[49] Humphrey S.P., Williamson R.T. (2001). A review of saliva: Normal composition, flow, and function. J. Prosthet. Dent..

[50] Roberts D.D., Acree T.E. (1995). Simulation of Retronasal Aroma Using a Modified Headspace Technique: Investigating the Effects of Saliva, Temperature, Shearing, and Oil on Flavor Release. J. Agric. Food Chem..

[51] Gonzales-Barron U., Butler F. (2008). Discrimination of crumb grain visual appearance of organic and non-organic bread loaves by image texture analysis. J. Food Eng..

[52] Aguayo-Mendoza M.G., Chatonidi G., Piqueras-Fiszman B., Stieger M. (2021). Linking oral processing behavior to bolus properties and dynamic sensory perception of processed cheeses with bell pepper pieces. Food Qual. Pref..

[53] Mokrzycki W.S., Tatol M. (2012). Colour difference DeltaE—A survey. Mach. Graph. Vis..

[54] Yven C., Patarin J., Magnin A., Labouré H., Repoux M., Guichard E., Feron G. (2012). Consequences of individual chewing strategies on bolus rheological properties at the swallowing threshold. J. Texture Stud..

[55] Drago S.R., Panouillé M., Saint-Eve A., Neyraud E., Feron G., Souchon I. (2011). Relationships between saliva and food bolus properties from model dairy products. Food Hydrocoll..

[56] Roger-Leroi V., Mishellany-Dutour A., Woda A., Marchand M., Peyron M.-A. (2012). Substantiation of an artificial saliva formulated for use in a masticatory apparatus. Odontostomatol. Trop..

[57] Leonor S.-P., Laura S.-M., Esther I.-C., Marco Z.-Z., Enrique A.-G.A., Ignacio M.-R. (2009). Stimulated saliva flow rate patterns in children: A six-year longitudinal study. Arch. Oral Biol..

[58] Schimmel M., Christou P., Herrmann F., Müller F. (2007). A two-colour chewing gum test for masticatory efficiency: Development of different assessment methods. J. Oral Rehabil..

[59] Tournier C., Rodrigues J., Canon F., Salles C., Feron G. (2015). A Method to Evaluate Chewing Efficiency in Infants Through Food Bolus Characterization: A Preliminary Study. J. Texture Stud..

[60] Jourdren S. (2017). Le Processus Oral, Une Etape Clé à L’origine des Propriétés Sensorielles de Texture et D’arôme du Pain. Ph.D. Thesis.

[61] Engelen L., de Wijk R.A., Prinz J.F., Janssen A.M., Weenen H., Bosman F. (2003). The effect of oral and product temperature on the perception of flavor and texture attributes of semi-solids. Appetite.

[62] Ben-Aryeh H., Fisher M., Szargel R., Laufer D. (1990). Composition of whole unstimulated saliva of healthy children: Changes with age. Arch. Oral Biol..

[63] Odake S., Roozen J.P., Burger J.J. (2000). Flavor Release of Diacetyl and 2-Heptanone from Cream Style Dressings in Three Mouth Model Systems. Biosci. Biotechnol. Biochem..

